# Screening and purification of NanB sialidase from *Pasteurella multocida* with activity in hydrolyzing sialic acid Neu5Acα(2–6)Gal and Neu5Acα(2–3)Gal

**DOI:** 10.1038/s41598-022-13635-x

**Published:** 2022-06-08

**Authors:** Christian Marco Hadi Nugroho, Ryan Septa Kurnia, Simson Tarigan, Otto Sahat Martua Silaen, Silvia Triwidyaningtyas, I. Wayan Teguh Wibawan, Lily Natalia, Andi Khomeini Takdir, Amin Soebandrio

**Affiliations:** 1 Doctoral Program in Biomedical Science, Faculty of Medicine, Universitas Indonesia, 10430 Jakarta, Indonesia; 2 Indonesian Research Center for Veterinary Science, RE Martadinata No. 30, 16124 Bogor, West Java Indonesia; 3 Animal Health Diagnostic Unit, PT Medika Satwa Laboratoris, Bogor, Indonesia; 4 Virology and Cancer Pathobiology Research Center, Faculty of Medicine, Universitas Indonesia, 10430 Jakarta, Indonesia; 5 Department of Animal Infectious Diseases and Veterinary Public Health, Faculty of Veterinary Medicine, IPB University, 16680 Bogor, Indonesia; 6 Department of Microbiology, Faculty of Medicine, Universitas Indonesia, 10320 Jakarta, Indonesia

**Keywords:** Drug discovery, Microbiology, Molecular biology

## Abstract

Study on sialidases as antiviral agents has been widely performed, but many types of sialidase have not been tested for their antiviral activity. *Pasteurella multocida* NanB sialidase is one such sialidase that has never been isolated for further research. In this study, the activity of NanB sialidase was investigated in silico by docking the NanB sialidase of *Pasteurella multocida* to the Neu5Acα(2–6)Gal and Neu5Acα(2–3)Gal ligands. Additionally, some local isolates of *Pasteurella multocida*, which had the NanB gene were screened, and the proteins were isolated for further testing regarding their activity in hydrolyzing Neu5Acα(2–6)Gal and Neu5Acα(2–3)Gal. Silico studies showed that the NanB sialidase possesses an exceptional affinity towards forming a protein–ligand complex with Neu5Acα(2–6)Gal and Neu5Acα(2–3)Gal. NanB sialidase of *Pasteurella multocida* B018 at 0.129 U/mL and 0.258 U/mL doses can hydrolyze Neu5Acα(2–6)Gal and Neu5Acα(2–3)Gal better than other doses. In addition, those doses can inhibit effectively H9N2 viral binding to red blood cells. This study suggested that the NanB sialidase of *Pasteurella multocida* B018 has a potent antiviral activity because can hydrolyze sialic acid on red blood cells surface and inhibit the H9N2 viral binding to the cells.

## Introduction

Avian influenza virus infection prevention and treatment have been frequently studied. Various types of vaccines and antiviral agents have been developed to reduce the severity of viral infection. Vaccination is performed to stimulate the development of a body's immune system that can neutralize the virus before it replicates^1^. Avian influenza virus is an RNA virus that allows the virus to mutate through an antigenic shift or antigenic drift quickly^2^. Mutation cause vaccination to be less effective because the antibodies formed cannot recognize the mutant viruses^3^.

On the other hand, the antivirals to treat avian influenza infection are also considered ineffective^4^. Various reports suggest that severe infections occur due to the inability of antivirals to inhibit the multiplication of viruses in cells adequately^5^. Resistance towards multiple classes of antivirals makes the virus difficult to contain^6^. A previous study showed that most avian influenza viruses isolated from human cases were resistant to oseltamivir and amantadine groups^7^.

Recently, various studies have been carried out to address the avian influenza virus infection problem, one of which is through sialidases^8^. The use of sialidases in inhibiting viral infection was first investigated by Malakhov et al.^9^ later known as DAS181^10^. Sialidase DAS181 is derived from the bacterium *Actinomyces viscosus*, part of the normal floras in human teeth. Further studies showed that sialidase DAS181 could prevent the death of 100% of the mouse population infected with the pathogenic H5N1 avian influenza virus and prevent the spread of infection in 70% of the total mice in a single population^10^. The success of DAS181 in suppressing the spread of avian influenza infection results in a prevailing interest in sialidases as antiviral agents. In 2010, sialidase from *Clostridium perfringens* bacteria from poultry was successfully isolated and was later known as sialivac. The administration of sialivac in avian influenza infection livestock outbreaks can prevent the spread of the virus infection^11^.

In overcoming avian influenza infection, each drug has a different mechanism of action. Generally, antivirals work after the virus enters the host cell, in contrast to sialidases which functions to degrade sialic acid (Neu5Ac) found on the surface of host cells. Sialidases prevent hemagglutinin (HA) protein from binding to sialic acid to perform endocytosis^12^. The unavailability of sialic acid is thought to disrupt the budding viral process after replication in the host cell, resulting in the inability of the virus to infect other cells^13^. Studies related to sialidases also showed a decrease in the severity of symptoms in chickens infected with avian influenza.

In addition to *Actinomyces viscosus* and *Clostridium perfringens*, the presence of sialidase was also detected in several other types of bacteria such as *Salmonella typhimurium*, *Vibrio cholerae*, *Corynebacterium diphtheria*, *Streptococcus pneumonia* to *Pasteurella multocida*^14–18^. Molecularly, these bacteria have genes encoding sialidase with different targets for sialic acid hydrolysis activity. For example, *Vibrio cholera* tends to hydrolyze Neu5Acα(2–3)Gal compared to Neu5Acα(2–6)Gal, whereas *Corynebacterium diphtheria* tends to hydrolyze Neu5Acα(2–6)Gal compared to Neu5Acα(2–3)Gal^16^.

*Pasteurella multocida* uses sialidase to obtain nutrients in hydrolyzed sialic acid^19^^.^ Of the many sialidase-producing bacteria, *Pasteurella multocida* is one with two genes encoding sialidase, namely NanH and NanB^14^. These genes encode sialidase protein which is a bacterial virulence factor in infecting the host. Several studies have screened the presence of NanH and NanB in *Pasteurella multocida* isolates and showed varied results, although the reason for these disparities is yet to be established^20^.

Although NanB and NanH are both sialidases derived from *Pasteurella multocida* bacteria, they have been shown to tend to hydrolyze different types of sialic acid. NanH tends to hydrolyze Neu5Acα(2–3)Gal, generally found in avian cells, while NanB sialidase is a broad-spectrum sialidase^19^. This is evidenced by the ability of NanB of *Pasteurella multocida* to hydrolyze Neu5Acα(2–3)Gal despite its resemblance to NanH of *Actinomyces viscosus,* which tends to hydrolyze Neu5Acα(2–6)Gal^21^.

Previous studies^19^ have shown the sialidase activity of *Pasteurella multocida* by expressing recombinant NanB sialidase in *Escherichia coli* bacteria, but the pure NanB sialidase protein has yet to be isolated. Therefore, in this study, in addition to predicting in silico the binding of NanB sialidase to Neu5Acα(2–6)Gal and Neu5Acα(2–3)Gal, authors screened the presence of the gene encoding sialidase in archival local isolates from Indonesia and characterize the NanB sialidase protein. Pure NanB sialidase is expected to hydrolyze sialic acid well so that it can be used in further research to study sialidase activity in inhibiting avian influenza infection.

## Materials and methods

### Bacteria, virus, NanB sialidase gene and ligand

Neu5Acα(2–6)Gal and Neu5Acα(2–6)Gal ligands were downloaded from the Protein Data Bank database web server (https://pubchem.ncbi.nlm.nih.gov) in 3D and optimized with Open Babel. The 3D structure of NanB sialidase from *Pasteurella multocida* isolate 86–1913 (Accession number AF274868) was obtained by entering the amino acid to Raptor X program. Then the molecular weight prediction of the NanB sialidase protein was carried out by entering the amino acid sequence at https://www.bioinformatics.org/sms/protmw.html. Meanwhile, to isolate pure NanB sialidase, screening was carried out on *Pasteurella multocida*, a local isolate archive of PT. Medika Satwa Laboratoris which only has one type of sialidase, namely NanB sialidase. Pure avian influenza subtype H9N2 strain A/Layer/Indonesia/WestJava04/2017 (MG957203) was used to show the relationship between the efficient hydrolysis of α2-6 and α2-3 bonds by NanB sialidase and anti-influenza activity.

### Analysis of the bonding of NanB sialidase *Pasteurella multocida* with Neu5Ac2-6Gal by in silico test

NanB and ligands were prepared using AutoDockTools 1.5.6 by removing water molecules and adding nonpolar hydrogens, charges, and atoms. The grid was arranged by making a gridbox that covers the target protein's surface followed with treatment by the auto grid program linked to the application. The molecular docking process of the ligand with NanB sialidase from *Pasteurella multocida* was carried out using the auto grid program. The docking output was the docked ligand's structure on the enzyme active site and its respective affinity score. Analysis of the docking results was carried out on residues interacting with the ligands, Gibbs free binding energy (∆G), structural conformation, affinity, and hydrogen bonding between NanB sialidase and the ligands^22^.

Visualization of molecular docking results between ligands and proteins was carried out using Edu PyMOL software. Visualization using Edu PyMOL software aimed to clarify the binding site of the ligand to the protein^22^.

### Culture, identification, and confirmation of *Pasteurella multocida*

A total of nine archival isolates were re-cultured using Brain Heart Infusion (BHI) Broth media (Himedia) and subsequently with Blood agar (BA) media (Himedia). Incubation was carried out overnight at 37 °C. The growing colonies were observed macroscopically and microscopically, followed by the catalase test^23^, oxidase test^24^, indole test^25^, and molecular confirmation through polymerase chain reaction (PCR) assay.

Pure colonies were extracted to obtain pure DNA using PrestoTM Mini gDNA Bacteria Kit (Geneaid). The primer used in the confirmation PCR test for *Pasteurella multocida* was an KMT1-specific primer pair with a target amplicon of 460 bp (Table 1). The PCR process was carried out using the KAPA2G Fast Hotstart Readymix PCR Kit (Merck) according to the relevant procedures with a final reaction volume of 10 μl and annealing temperature optimized for 55 °C for 40 cycles. The amplified sample was then visualized by electrophoresis using 1.5% agarose gel (1st base) and stained using 0.5 g/ml ethidium bromide (Vivantis). The marker used was 100 bp (VC 100 bp Plus DNA Ladder Vivantis) as a standard measure. Isolates identified as *Pasteurella multocida* through PCR testing were then tested on the *hyaD-hyaC* and *bcbD* genes (Table 1) to differentiate the identified serotypes of *Pasteurella multocida*^27^.

**Table 1:** List of Primers for Identification of *Pasteurella multocida*.

Genes	Sequences	Amplicon (bp)
KMT1^27^	T7(F) 5′-GCGTTTCATTCAAAGCATCTC-3′	460
	SP6(R) 5′-ATGACCGCGTAACGACTTTC-3′	
*hyaD-hyaC*^28^	CapA(F) 5′-TGCCAAAATCGCAGTGAG-3′	1044
	CapA(R) 5′-TTGCCATCATTGTCAGTG-3′	
*bcbD*^28^	CapB(F) 5′-CATTTATCCAAGCTCCACC-3′	758
	CapB(R) 5′-GCCCGAGAGTTTCAATCC-3′	
NanB^29^	(F) 5′-GTCCTATAAAGTGACGCCGA-3′	554
	(R) 5′-ACAGCAAAGGAAGACTGTCC-3′	
NanH^29^	(F) 5′-GAATATTTGGGCGGCAACA-3′	360
	(R) 5′-TTCTCGCCCTGTCATCACT-3′	

### Identification and molecular characterization of sialidase-coding genes *Pasteurella multocida*

Identification of the presence of the gene encoding *Pasteurella multocida* sialidase was carried out on isolates that had been identified as *Pasteurella multocida* type A. Screening for the presence of the sialidase gene was carried out using the NanB and NanH primer (Table 1). The KAPA2G Fast Hotstart Readymix PCR Kit (Merck) was used in the PCR process with a total PCR reaction of 50 μl and an annealing temperature of 56 ℃^30^. The sequencing of amplicons that only showed positive NanB were performed by 1st base sequencing service agency, Malaysia. Sequencing data were analyzed using MEGA X and Bioedit software to compare the genetics of the study isolate NanB with the NanB gene belonging to *Pasteurella multocida* 86-1913.

### Native production of NanB sialidase *Pasteurella multocida*

*Pasteurella multocida* bacteria containing only the gene encoding NanB sialidase was propagated in the main batch of Brain Heart Infusion (BHI) broth (Himedia) 1000 ml and then centrifuged at 4 °C at a speed of 4000×*g* for 45 min. The bacterial pellet was used for the subsequent production of NanB sialidase^31^.

Some of the methods used are the chloroform method with a slight modification^32^, the glycine method^33^, freeze-thaw^34^, and osmotic shock^35^. In this study, several modifications of osmotic shock were used to divide them into the original method, the addition of Ca^2+^, the addition of lysozyme, and a combination of Ca^2+^ and lysozyme. Based on the results of sialidase production, the method that gave the highest specific activity value of sialidase was used as the sialidase production method, followed by anion exchange chromatography and affinity chromatography.

Anion exchange chromatography was performed using Q-sepharose with the resulting fractions labeled as F0, F1, F2, F3, F4, and F5, respectively. The fraction showing the highest specific activity was purified by column (5 ml) affinity chromatography using N-(p-Aminophenyl)oxamic acid–Agarose (Sigma-Aldrich) according to the relevant procedures. The estimated molecular weight of protein sialidase was carried out using SDS PAGE at 12% separating gel and 4% stacking gel^36^. At each stage of purification, protein count was performed using the Bradford method and tested for sialidase activity using the Neuraminidase assay kit (Sigma-Aldrich) according to the relevant procedures.

### Optimum pH, temperature and sialidase incubation period tests

To find the optimum temperature, 80 μl of the Neuraminidase assay kit (Sigma-Aldrich) reaction mixture and 20 μl of NanB sialidase protein fraction were mixed and then incubated at different temperatures, namely 20, 25, 30, 37, 40, 45 and 50 °C. The sialidase activity was subsequently calculated^37^.

The determination of the optimum pH was tested by incubating 20 µl of sialidase in pH 3 to pH 10 (1:1) in 80 µl of the Neuraminidase assay kit (Sigma-Aldrich) reaction mixture at 37 °C. The sialidase activity was subsequently calculated as Units/ml in the same manner as the previous protocol. Meanwhile, the determination of sialidase activity in a certain incubation period was carried out by incubating sialidase at 37 °C and then calculating its activity at 24, 48, and 72 h of incubation^37^.

### Sialidase toxicity test on red blood cells

NanB sialidase toxicity test was carried out on chicken and rabbit red blood cells. A total of 500 μl of sialidase in graded doses (0 U/mL, 0.032 U/mL, 0.064 U/mL, 0.129 U/mL and 0.258 U/mL) was added to 500 μl of chicken and rabbit red blood cells with a concentration of 1% each and then incubated at 37 °C for 2 h. Centrifugation was carried out at a speed of 6000×*g* for 3 min, then transferred the supernatant to a microplate and read using a microplate reader with a wavelength of 562 nm. As a lysis control, lysis buffer (Geneaid) was used. Toxicity was calculated using the following formula: 100 × (OD of sample—OD of negative control/ OD of lysis control—OD of negative control)^38^.

### Sialidase specificity test for sialic acid in chicken and rabbit red blood cells

Red blood cells of chickens and rabbits were treated with the addition of graded sialidase concentrations (0 U/mL, 0.032 U/mL, 0.064 U/mL, 0.129 U/mL and 0.258 U/mL) for 2 h at 37 °C. Cells were washed three times with PBS and fixed with 0.05% glutaraldehyde in PBS. Cell-based enzyme-linked lectin assay measured the amounts of (2,6)-linked sialic acid and (2,3)-linked sialic acid. The fixed cells were blocked with 3% bovine serum albumin (BSA) in PBS and streptavidin–biotin blocking reagent (Vector Laboratories, Burlingame, CA) to block endogenous streptavidin- and biotin-binding sites. Cells were rinsed once with PBS-0.1% Tween 20 (PBST) and incubated with 2 µg biotinylated SNA lectin (Vector Laboratory)/ml and 20 µg biotinylated MAA lectin (Vector Laboratory)/ml at 37 °C. SNA (Sambucus nigra) is specific for Neu5Acα(2–6)Gal while MAA (Maackia amurensis) is specific for Neu5Acα(2–3)Gal. The cells were washed four times with PBST. Secondary detection of bound lectins was carried out by incubating 5 g streptavidin-HRP/ml for 1 h at 37 °C. Cells were washed five times in PBST, added in tetramethylbenzidine (TMB; Sigma), and suspended in 1 M H_2_SO_4_. The absorbance was measured at 450 nm, and the percentage of sialic acid remaining was calculated using the following calculation: 100% × [(absorbance of treated cells − background)/(absorbance of treated cells − background)]. Cells treated with streptavidin-HRP alone without lectins were used as a background control^9^.

### H9N2 virus binding inhibition test on post- red blood cells administration of sialidase

The H9N2 virus binding inhibition test on red blood cells was carried out in six repetitions with graded sialidase concentrations (0 U/mL, 0.032 U/mL, 0.064 U/mL, 0.129 U/mL and 0.258 U/mL). A total of 500 μl of graded sialidase concentration was added to 500 μl of 1% concentration of chicken and rabbit red blood cells and then incubated at 37 °C for 2 h. After incubation, 500 μl 9 log 2 HAU of avian influenza H9N2 virus was poured into a mixture of sialidase and red blood cells and then incubated for 30 min at room temperature. Then centrifuged at 5000 rpm for 5 min. The supernatant was discarded and the pellet in the form of red blood cells was extracted by RNA using the Total RNA Mini Kit (Geneaid) and then followed by qRT-PCR to count the number of RNA copies of the virus that were still bound to the red blood cells. The qRTPCR process was carried out using the Sensifast SYBR Lo-ROX One-Step Kit (Bioline) kit according to the protocol for use.

### Statistical analysis

Data were analysed in Graphpad Prism 9.1.2. The mean and standard error of the mean (SEM) or standard deviation (SD) were used to display normally distributed data. The data comparison between the groups were analysed using analysis of variance (ANOVA).

### Ethics declarations

No experimental animals were used in this study, however this study has carefully reviewed and approved by The Ethics Committee of the Faculty of Medicine, University of Indonesia (No. KET-1215/UN2.F1/ETIK/PPM.00.02/2020).

## Results

### In silico bonding of NanB sialidase *Pasteurella multocida* with Neu5Acα(2–6)Gal ligand

**Figure 1:**
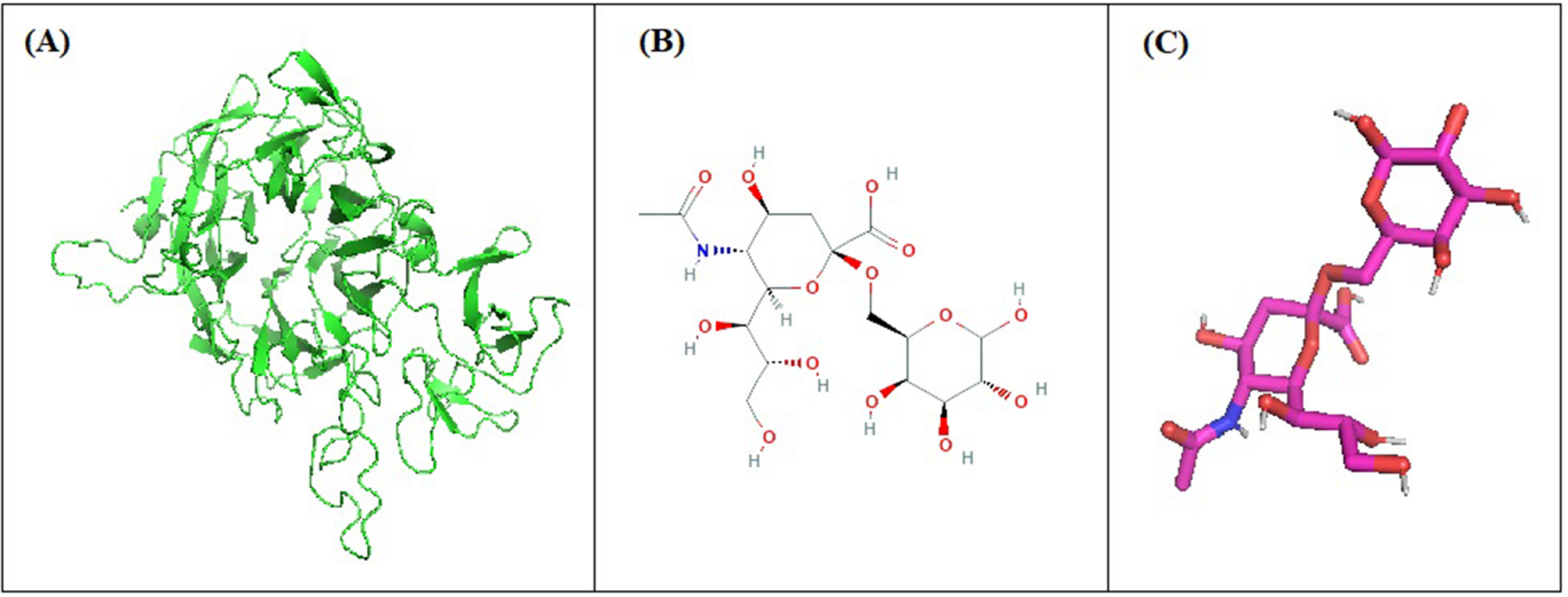
Some of the structures required in the docking process. (**A**) NanB sialidase protein generated from the Raptor X program; (**B**) 2D structure of Neu5Acα(2–6)Gal; (**C**) Neu5Acα(2–6)Gal 3D structure.

**Figure 2:**
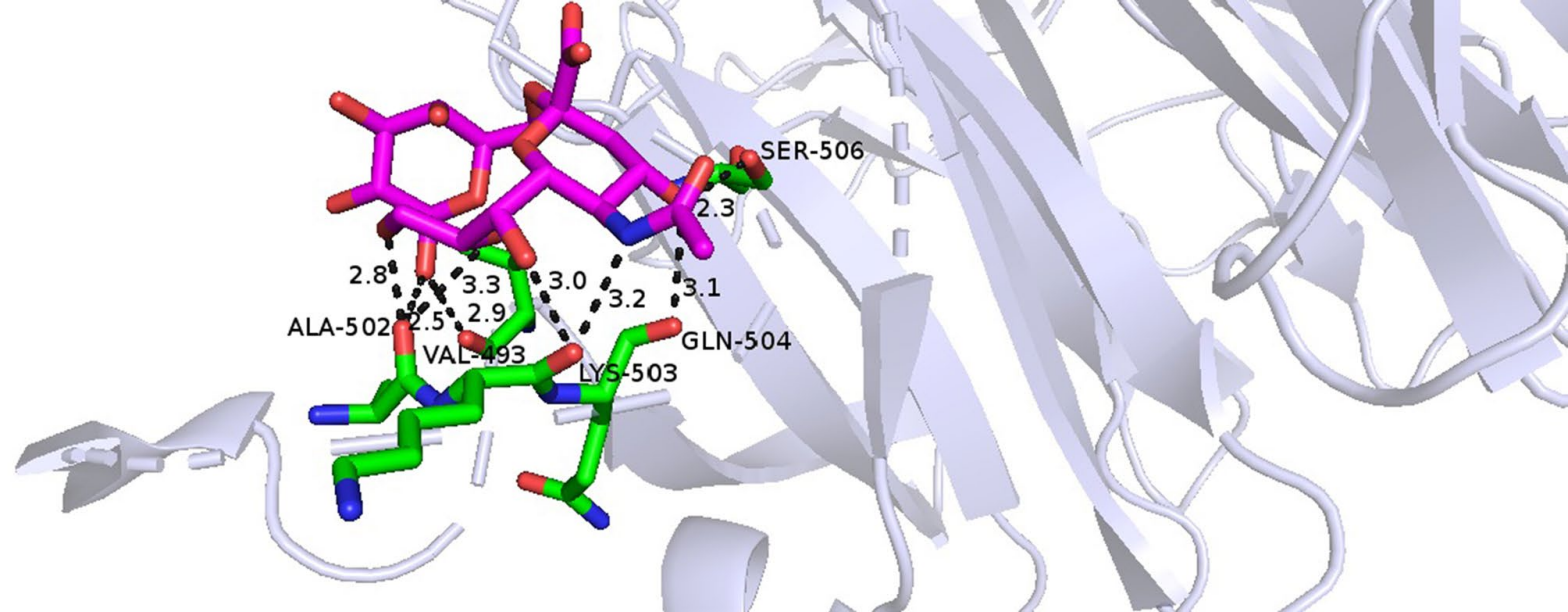
Visualization of interactions between Neu5Acα(2–6)Gal and NanB sialidase using Pymol showing hydrogen bonds, residues and their interaction distance with Neu5Acα(2–6)Gal in 3D.

**Figure 3:**
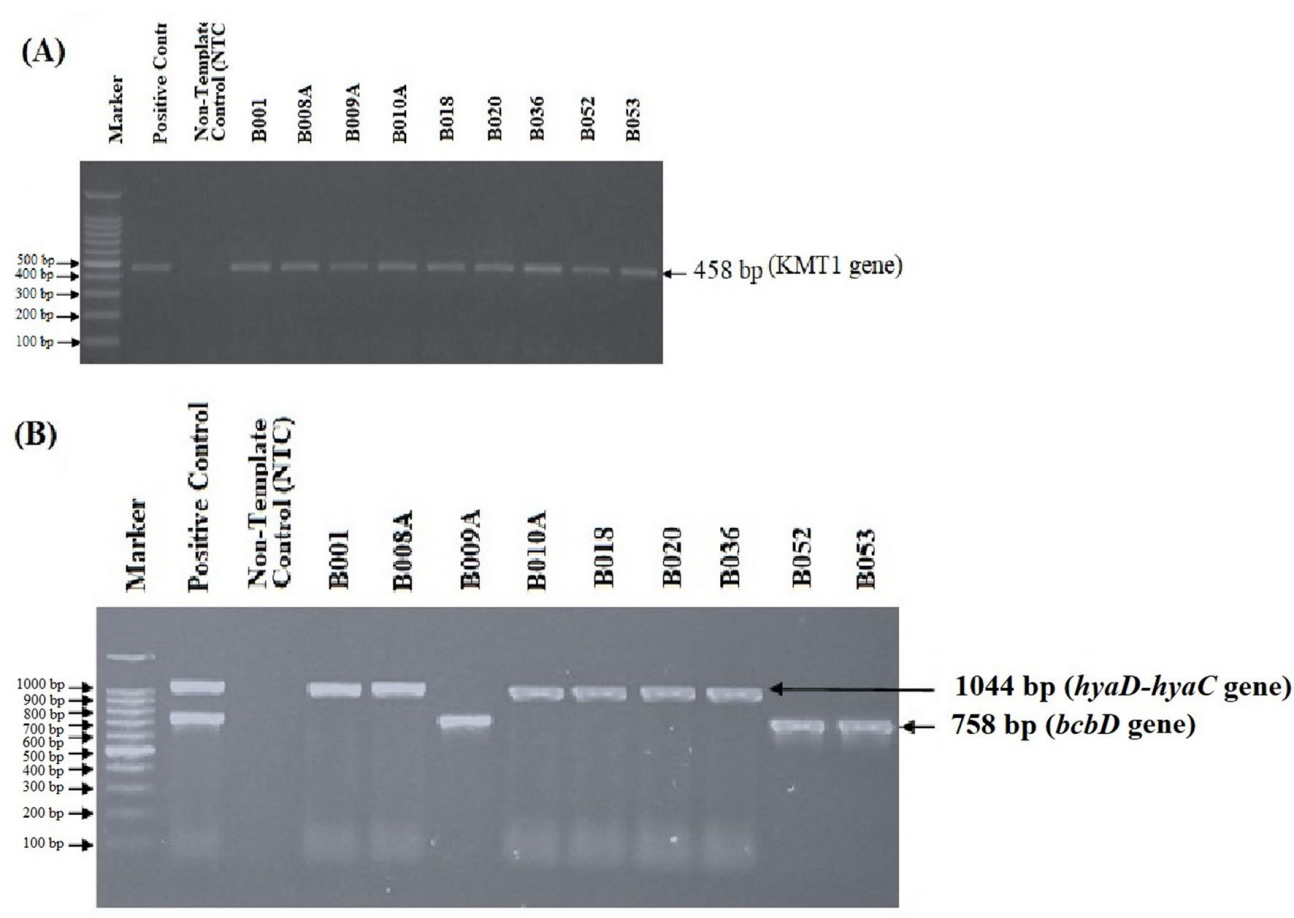
PCR test results confirm *Pasteurella multocida* and its serotypes. (**A**) amplification of the ompH gene from all isolates showed a position of 460 bp; (**B**) PCR test results to distinguish serotypes from *Pasteurella multocida*. The positive control is the result of DNA extraction from locale isolates of *Pasteurella multocida* types A and B mixed that showed two bands of the gel electrophoresis.

The 3D structure of NanB sialidase from *Pasteurella multocida* was generated from the Raptor X program (Fig. 1A), while the Neu5Acα(2–6)Gal ligand was successfully prepared from the 2D form (Fig. 1B) to 3D form (Fig. 1C) with the Open Babel program. Based on the results of the formation of 3D NanB sialidase, there were differences in the number of amino acid sequences from 1070 amino acids to 503 amino acids. These results also affect the prediction of the molecular weight of the NanB sialidase protein which https://www.bioinformatics.org/sms/prot_mw.html analyzed. The results of the analysis of the amino acid composition of Raptor X obtained that the molecular weight of NanB sialidase was 56.44 kDa, very different from the initial amino acid prediction of 119.81 kDa.

Based on the docking results between NanB sialidase and the Neu5Acα(2–6)Gal ligand, the Gibbs free energy or ∆G of − 5.56 kcal/mol was obtained. While Ki value produced by the recent docking process in this study was 84.26 μM. This was reinforced by the conformation between the ligand binding and the receptor, indicating that the Neu5Acα(2–6)Gal and NanB sialidase ligands can bind to each other. In this study, the hydrogen bonding interaction of NanB sialidase with Neu5Acα(2–6)Gal was shown at Val 493, Ala 502, Lys 503, Gln 504, Ser 506 with an overall distance below 3.9 Å (Fig. 2).

NanB sialidase and the Neu5Acα(2–3)Gal ligand showed − 7.15 kcal/mol for the Gibbs free energy value or ∆Gand the Ki value was 5.70 μM. There were some hydrogen bonding interactions of NanB sialidase with Neu5Acα(2–3), including Arg69, Gln169 and Gly321.

### Culture, identification, and confirmation of *Pasteurella multocida*

Identification of *Pasteurella multocida* was carried out using macroscopic, microscopic, and biochemical tests, further continued by the PCR test (Table 2). The results showed that all isolates were *Pasteurella multocida* bacteria (Fig. 3A), while the results of type classification through PCR showed that only six of the nine isolates were *Pasteurella multocida* type A (Fig. 3B).

**Table 2:** Results of culture, identification and confirmation of *Pasteurella multocida* with various test methods.

No	Isolate	Macroscopic	Microscopic	Catalase test	Oxidase test	Indole test	PCR test (ompH)	Multiplex PCR test	
								CapA	CapB
1	B001	+	+	+	+	+	+	+	−
2	B008A	+	+	+	+	+	+	+	−
3	B009A	+	+	+	+	+	+	−	+
4	B010A	+	+	+	+	+	+	+	−
5	B018	+	+	+	+	+	+	+	−
6	B020	+	+	+	+	+	+	+	−
7	B036	+	+	+	+	+	+	+	−
8	B052	+	+	+	+	+	+	−	+
9	B053	+	+	+	+	+	+	−	+

### Identification and molecular characterization of sialidase *Pasteurella multocida*

**Figure 4:**
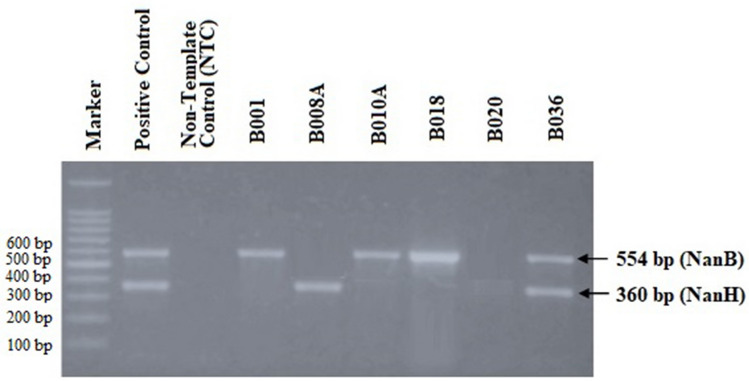
The detection results of NanB and NanH sialidase genes from six isolates confirmed as *Pasteurella multocida* type A. NanB is indicated by the 554 bp, while NanH in the 360 bp.

Molecular identification of the gene encoding sialidase was carried out on six isolates of *Pasteurella multocida*, previously confirmed as type A. The amplicon position was shown at 554 bp for isolates identified as having NanB sialidase and 360 bp for isolates having NanH sialidase. In this study, the isolates tested against the two genes gave varied results. Namely, three isolates only had NanB sidalise, one isolate only had nanH sialidase, one isolate had both types of sialidase, and another isolate did not have the gene encoding sialidase (Fig. 4).

**Figure 5:**
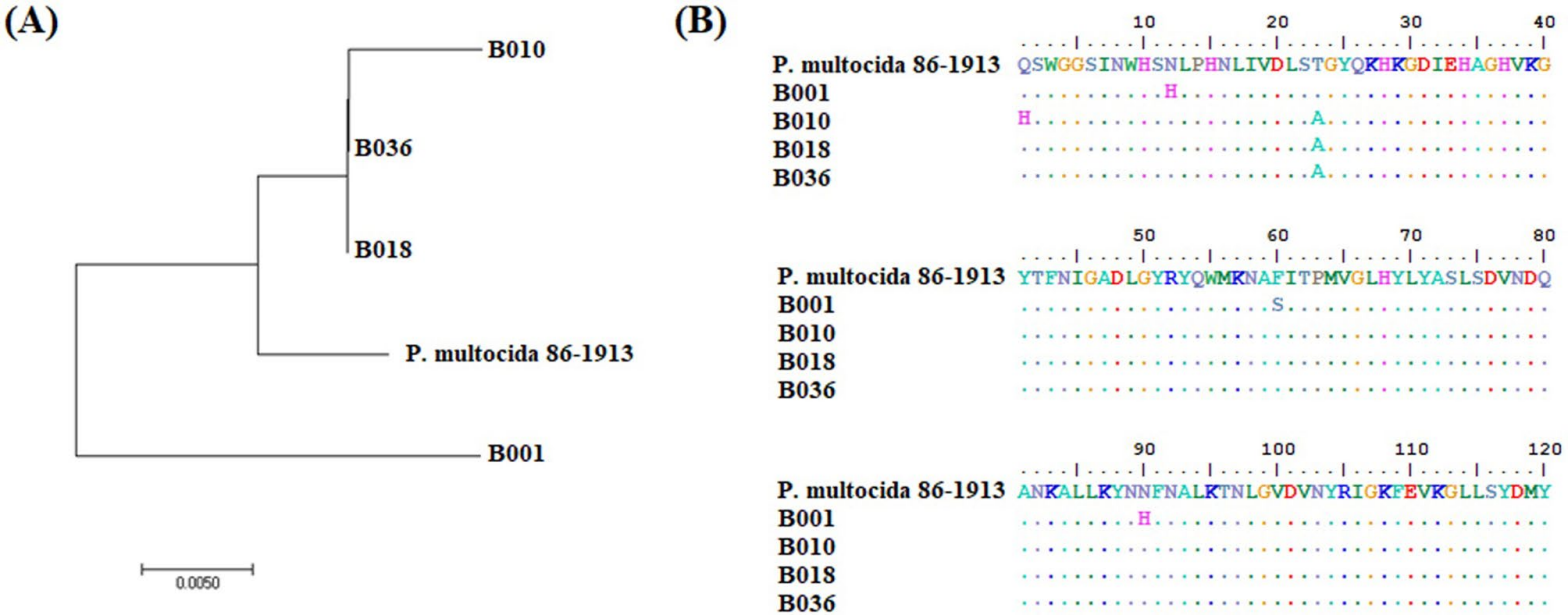
Results of the *Pasteurella multocida* isolate selection test to be used in purification of protein sialidase. (**A**) Phylogenetic tree showing the relationship between *Pasteurella multocida* isolates; (**B**) Alignment of amino acids with the Bioedit program to determine points of difference in amino acids.

Based on the similarity of amino acid sequence and close kinship to isolate *Pasteurella multocida* 86–1913, some isolates in this study were selected for the isolation of NanB sialidase (Fig. 5). Isolate B018 was chosen because it has a single sialidase NanB (Fig. 4) and shows the highest amino acid sequence similarity, 99.00% with *Pasteurella multocida* 86–1913 (Table 3).

**Table 3:** The results of the test of the similarity level of the NanB gene isolate with *Pasteurella multocida* 86–1913 as a reference isolate.

Strain isolates		1	2	3	4	5
1	*Pasteurella multocida* 86–1913					
2	B001	96.76				
3	B010	98.40	96.33			
4	B018	99.00	96.96	99.40		
5	B036	99.00	96.96	99.40	100	

### Isolation and purification of protein NanB sialidase *Pasteurella multocida* B018

Among the various isolation methods carried out, the results showed that the chloroform method could produce the highest specific activity of NanB sialidase, namely 0.202 U/mg (Table 4). Some methods that were also quite good in providing specific activity of NanB sialidase were Glycine and Freeze–thaw with specific activity values of 0.191 U/mg and 0.152 U/mg. In contrast to the other three methods, the osmotic shock method gave relatively lower values, both for the first supernatant containing sucrose, Tris–HCl, and EDTA, and the second supernatant in the form of reverse osmosis water (Table 4).

**Table 4:** Results of testing the specific activity of NanB sialidase from various isolation methods.

NanB sialidase isolation method	Supernatant	Supernatant volume (ml)	Protein concentration (mg/ml)	Total protein (mg)	Sialidase activity (U/ml)	Total sialidase activity (U)	Specific activity (U/mg)
Kloroform	Tris–HCl pH 8.0	1	0.823	0.823	0.166	0.166	0.202
Glysin	Sterile aquades + Glysin 1%	1	0.497	0.497	0.095	0.095	0.191
Freeze thaw	Potasium phosphat buffer pH 6.8	1	0.611	0.611	0.093	0.093	0.152
**Osmotic shock**							
a. Original	sucrose, Tris–HCl, EDTA	1	0.819	0.819	0.089	0.089	0.109
	reverse osmosis (RO) water	1	0.203	0.203	0.002	0.002	0.009
b. Addition of Ca^2+^	sucrose, Tris–HCl, EDTA	1	0.870	0.870	0.075	0.075	0.087
	reverse osmosis (RO) water	1	0.249	0.249	0.008	0.008	0.034
c. Addition of lysozyme	sucrose, Tris–HCl, EDTA	1	0.893	0.893	0.081	0.081	0.091
	reverse osmosis (RO) water	1	0.198	0.198	0.013	0.013	0.067
d. Combination of Ca^2+^ and lysozyme	sucrose, Tris–HCl, EDTA	1	0.816	0.816	0.068	0.068	0.083
	reverse osmosis (RO) water	1	0.216	0.216	0.021	0.021	0.099

Crude NanB sialidase, which was isolated by chloroform method, was subsequently treated with anion exchange chromatography. An unusual result was observed at this stage: a decrease in the target protein at F0, which showed the highest sialidase-specific activity of 1.79 U/mg (Table 5). In this study, anion exchange chromatography and affinity chromatography increased purity of 6.9 and 40.13 times compared to crude NanB sialidase. The overall purification results can be seen in Table 6.

**Table 5:** Results of purification of NanB sialidase by anion exchange chromatography.

Anion exchange chromatography fraction	Supernatant volume (ml)	Total protein (mg)	Total sialidase activity (U)	Specific activity (U/mg)
C*crude* protein	100	64.80	16.80	0.259
F0	100	8.60	15.40	1.790
F1 (0.2 M NaCl)	5	10.66	0.14	0.013
F2 (0.4 M NaCl)	5	11.13	0.02	0.002
F3 (0.6 M NaCl)	5	1.82	0.01	0.006
F4 (0.8 M NaCl)	5	0.02	0.001	0.023
F5 (1 M NaCl)	5	0.03	0.001	0.020

**Table 6:** Results of purification of NanB sialidase by anion exchange chromatography and affinity chromatography.

NanB sialidase isolation method	Supernatant volume (ml)	Total protein (mg)	Total sialidase activity (U)	Specific activity (U/mg)	Sialidase activity (U/ml)	Purification fold
Crude protein	100	64.80	16.80	0.259	0.168	1
Anion exchange chromatography	100	8.60	15.40	1.791	0.154	6.9
Affinity chromatography	15	0.372	3.87	10.40	0.258	40.13

**Figure 6:**
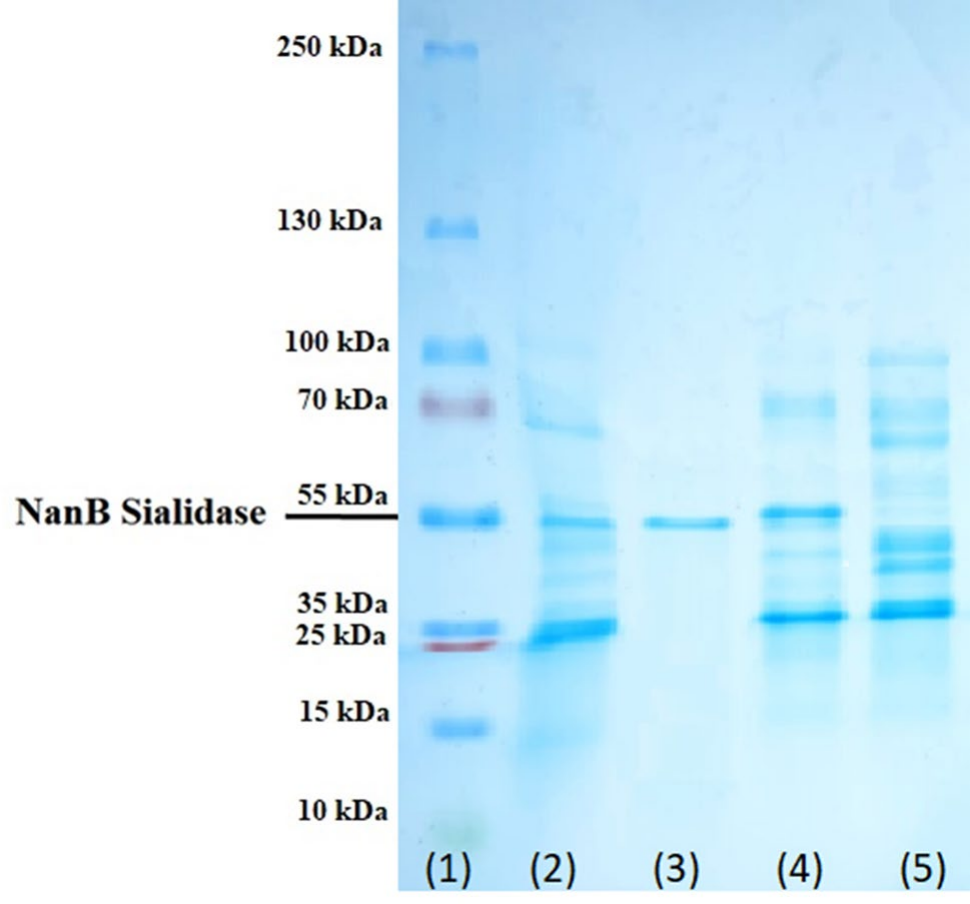
Results of SDS Page on protein at each stage of NanB sialidase *Pasteurella multocida* B018 purification. (1) Markers; (2) crude sel of *Pasteurella multocida* before chloroform method; (3) protein after Affinity chromatography; (4) protein after anion exchange chromatography; (5) initial protein supernatant crude sialidase.

The molecular weight measurements of NanB Sialidase produced in this study are novelties that have not been reported in previous studies correctly. NanB sialidase produced from *Pasteurella multocida* B018 showed a size of about 55 kDa (Fig. 6).

### Optimum temperature, pH and incubation period for NanB sialidase *Pasteurella multocida* B018

**Figure 7:**
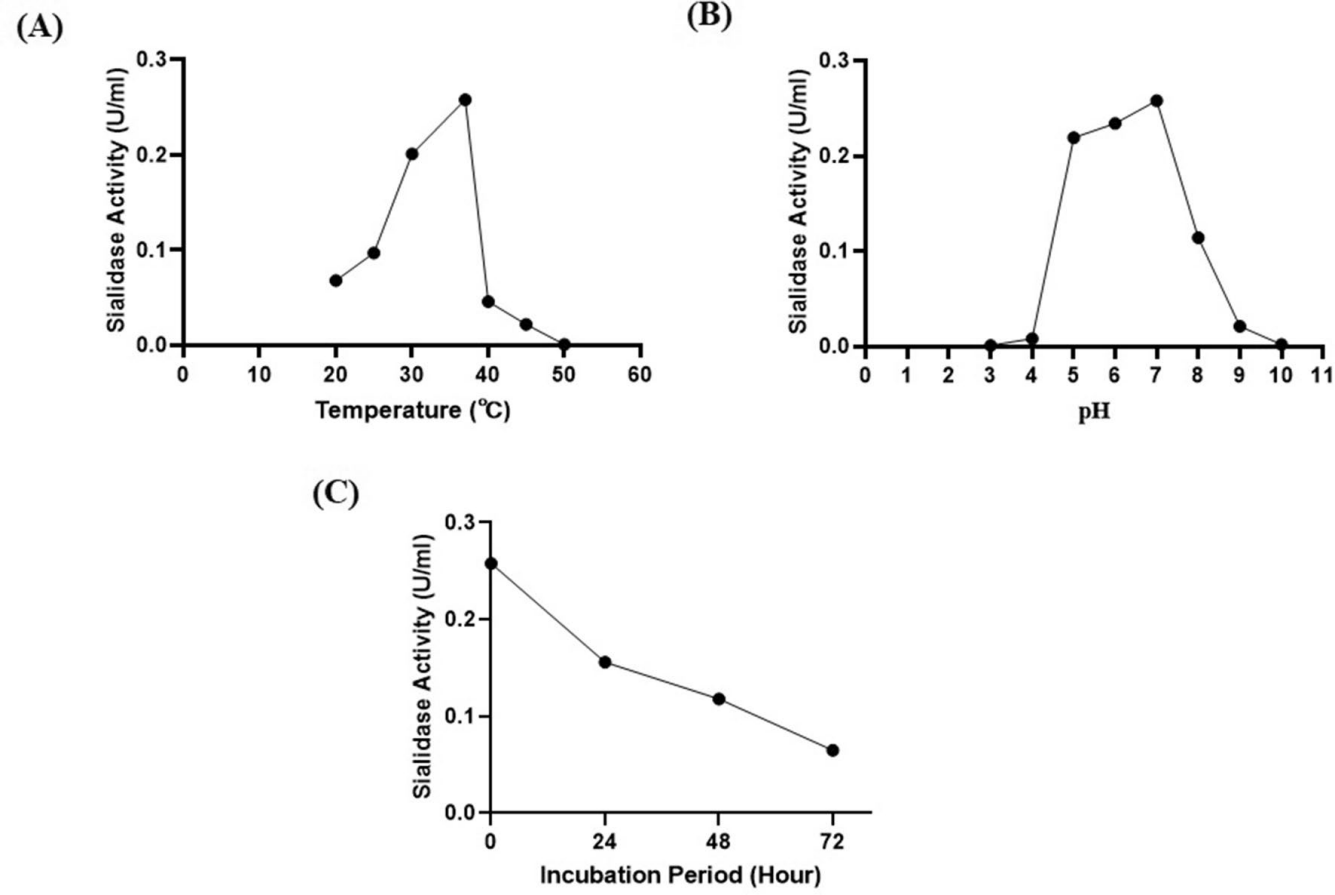
Test results for optimum temperature, pH and incubation period of NanB sialidase *Pasteurella multocida* B018. (**A**) Optimum temperature; (**B**) optimum pH; (**C**) incubation period.

The optimum temperature in this study is defined as the temperature that shows the highest sialidase activity and stability. A temperature of 37 °C is an ideal condition for several enzymes, including NanB sialidase. Reasonably good results were also shown at the incubation temperature of 30 °C. In contrast to the two temperatures, sialidase activity was less than optimum at 20 °C and 25 °C and even showed deficient activity at 40–50 °C (Fig. 7A).

Tests on the optimum pH of sialidase also showed quite varied results. The optimum sialidase activity was indicated by pH 7, followed by pH 5 and 6, which were still quite good in producing sialidase activity. At pH 3 and 4, the sialidase activity decreased to zero (0). At pH 8 showed lower sialidase activity than pH 7, and pH 9–10 produced deficient sialidase activity (Fig. 7B).

After incubation for 24 h, testing of sialidase activity showed a decrease in activity from 0.258 U/ml to 0.156 U/ml. The same results were also shown at 48 and 72 h of incubation, which decreased activity to 0.118 and 0.065 U/ml. Sialidase activity decreased when incubated at 37 °C for 72 h (Fig. 7C).

### NanB sialidase toxicity test results *Pasteurella multocida* B018

**Figure 8:**
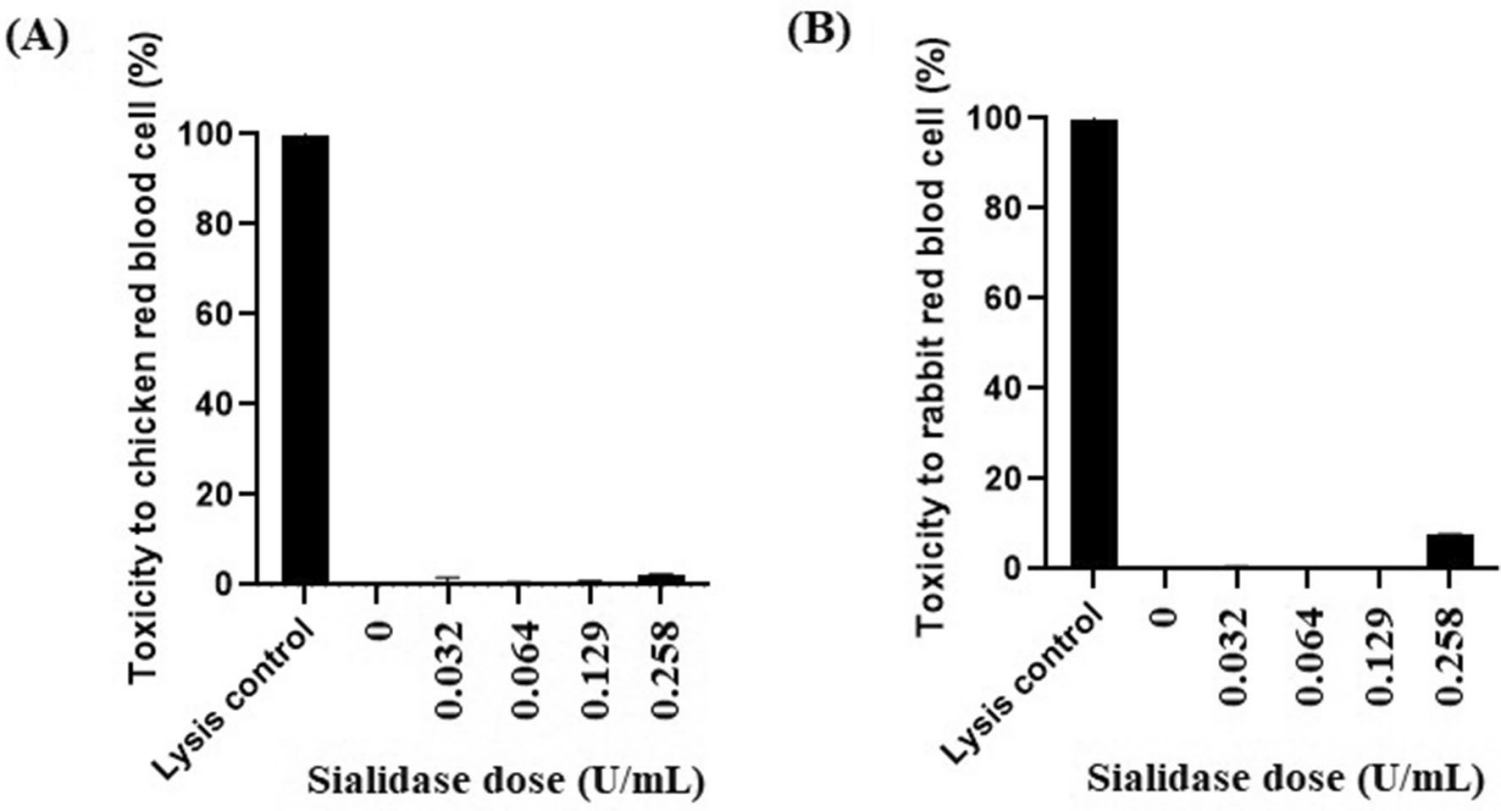
Results of sialidase toxicity test on red blood cells of chicken and rabbit. (**A**) chicken red blood cells; (**B**) rabbit red blood cells.

The results showed that the 0.258 U/mL sialidase dose caused the lysis of red blood cells in chickens 2.12% (Fig. 8A) as much as rabbits 7.65% (Fig. 8B). Both results represent the highest level of red blood cell damage compared to several other doses. Tests with a 0.129 U/mL dose of sialidase showed a much lower level of toxicity, namely 0.74% lysed chicken red blood cells, and 0.46% lysed rabbit red blood cells. Similarly, the two doses of sialidase, namely 0.064 U/mL and 0.032 U/mL, showed 0.42% lysis of chicken red blood cells and 0.31% and 0.08% lysis of rabbit red blood cells, respectively.

### Sialidase specificity test for sialic acid in chicken and rabbit red blood cells

**Figure 9:**
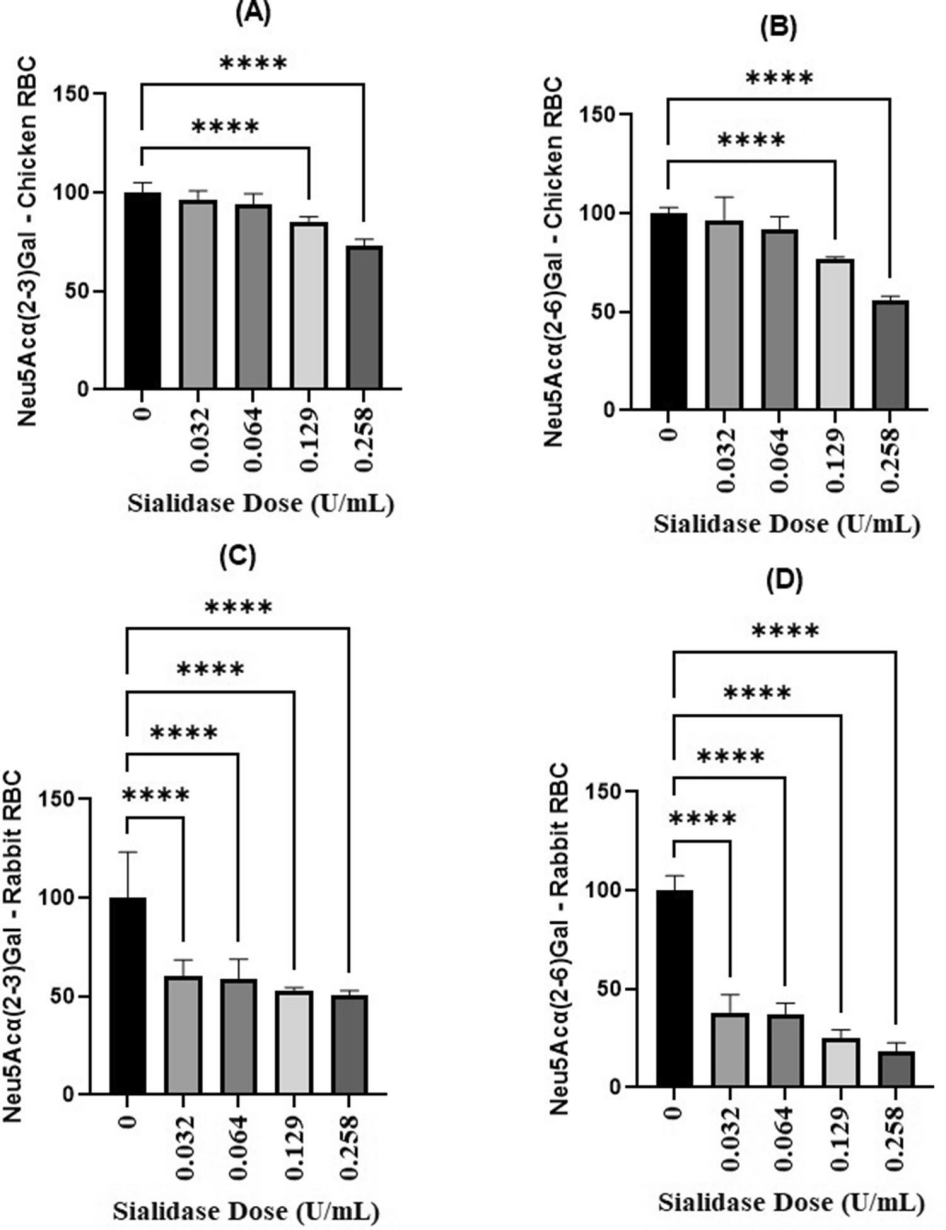
Results of NanB sialidase specificity test for two different sialic acids and two different blood types. (**A**) Neu5Acα(2–3)Gal, chicken red blood cells; (**B**) Neu5Acα(2–6)Gal, chicken red blood cells; (**C**) Neu5Acα(2–3)Gal, rabbit red blood cells; and (**D**) Neu5Acα(2–6)Gal rabbit red blood cells.

This study succeeded in demonstrating the activity of sialidase in hydrolyzing two sialic acids, namely Neu5Acα(2–6)Gal and Neu5Acα(2–3)Gal. This is evidenced by the reduced amount of these two types of sialic acid in the red blood cells of chickens and rabbits (Fig. 9).

The calculation of residual sialic acid in chicken blood showed that there was a meaningful difference in the percentage of residual sialic acid between the control (0 U/mL) and the treatment group with the percentage of sialidase 0.129 U/mL and 0.258 U/mL (p < 0.05). In contrast, the residual amount of sialic acid in rabbit blood showed a meaningful difference in percentage between the control (0 U/mL) and the entire sialidase dose group (p < 0.05).

### Inhibition of H9N2 virus binding on red blood cells after administration of sialidase

**Figure 10:**
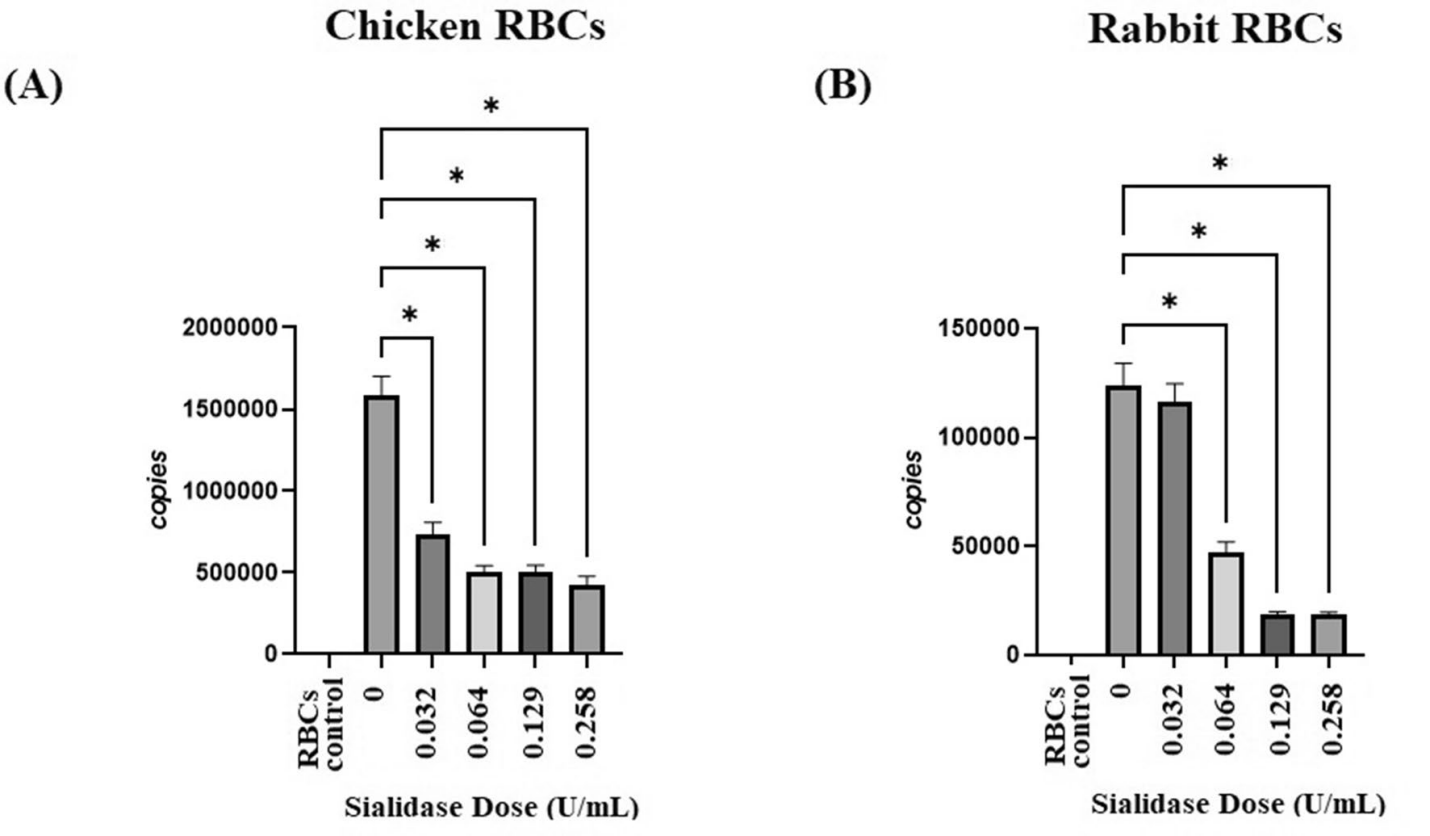
Viral copy number of H9 genes of the viruses that bind to red blood cells. (**A**) chicken red blood cells and (**B**) rabbit red blood cells.

Quantitatively, the binding inhibition of the H9N2 subtype of avian influenza virus on red blood cells was assessed based on the copy number of the H9 virus gene bound to chicken and rabbit red blood cells through RT-qPCR. The results showed that there were differences in the copy number of the virus in chicken red blood cells, indicated by all treatment groups the percentage of sialidase (0.032 U/mL, 0.064 U/mL, 0.129 U/mL and 0.258 U/mL) when compared to the control (0 U/ml). With p value < 0.05. Meanwhile, in rabbit red blood cells, three doses were significantly different (p < 0.05) compared to control (0 U/ml), namely 0.064 U/ml, 0.129 U/ml and 0.258 U/ml with the best dose of 0.129 U/ml and 0.258 U/ml (Fig. 10).

## Discussion

NanB sialidase is the receptor used as a docking material in this study. The enzyme was derived from *Pasteurella multocida* isolate 86–1913 available at GenBank with the access code AF274868 and has never been crystallized. As such, it required 3D structure prediction via Raptor X. The selection of docking results in this study was based on results that showed complete protein binding and density. X-Ray (RMSD) detected a value of 2 Å for docking of NanB sialidase and the ligands^39^.

Although only an approximation, − 5.56 kcal/mol for Neu5Acα(2–6)Gal and -7.15 kcal/mol for Neu5Acα(2–3)Gal of ∆G are welcome results. A negative ∆G value indicates a spontaneous formation of the protein–ligand complex due to the stability and strength of the noncovalent interactions found in the complex. Experimentally ∆G is directly related to the Ki. Thus, the value of ∆G can be used to approximate the ability of a compound to inhibit the protein. The Ki value produced by recent docking is the lowest of several docking results produced. Together with ∆G, the value of Ki determines the affinity value. The lower these two values are, the higher the affinity of the docked ligand and the more stable the bond that occurs^40^.

In addition to ∆G and pKi, hydrogen bonding parameters can also be used to evaluate the binding affinity of the complex formed between the receptor and the ligand^41^. Hydrogen bonding is the most important specific interaction in the process of interaction between ligands and receptors. Therefore, hydrogen bonding contributes to the molecule's affinity for the target protein that forms an electrostatic interaction between the hydrogen donor and acceptor^42^. In a hydrogen bond interaction analysis, the criteria for hydrogen bonding require a hydrogen donor and acceptor with a bond distance of 3.9 Å^43^. The number of hydrogen bonds produced by pairing the enzyme NanB sialidase and the Neu5Acα(2–6)Gal ligand supports the notion that the two have an excellent binding affinity. Based on the suitability of several parameters analyzed by docking, the ability of NanB sialidase to hydrolyze sialic acid was tested directly through true-experimental laboratory testing.

Identification of Sialidase in this study was carried out using two primer pairs NanB and NanH, as carried out previously by^29^. Based on the result of PCR, *Pasteurella multocida* in this study showed various NanB and NanH sialidase genes combination. Similar results were previously reported by Gharibi et al.^20^ 63.6% of the studied isolates had NanB sialidase, and 81.8% had the gene encoding NanH sialidase. This finding was similarly reported in previous studies by Furian et al.^44^ and Khamesipour et al.^45^. Although it is known that sialidase is an enzyme that removes sialic acid conjugated with glycolipids and glycoproteins of eukaryotic cells^46^, the cause of genetic variation in the presence of NanB and NanH genes from each strain of *Pasteurella multocida* is not yet established.

In contrast to NanH sialidase, which can be quickly released from *Pasteurella multocida*, NanB sialidase is more challenging to isolate. This is presumably due to NanB sialidase being a transmembrane protein firmly bound to the outer cell membrane to the periplasm of the bacterium *Pasteurella multocida*^19^. Mizan et al.^19^ concluded that NanB sialidase is one of the proteins with an autotransporter part because it has a channel-forming C-terminal domain that is thought to be involved in protein translocation from the bacterial outer membrane. However, this study has not succeeded in isolating the sialidase released directly on the supernatant of the bacterial growth medium. Therefore, optimization of the method was carried out to determine which method could produce the highest sialidase activity.

The chloroform method was first introduced as a periplasmic protein extraction method by Ames et al.^32^ who stated that the chloroform method could release 16% of the total cell protein and did not damage the activity of the released protein. The same method was also applied by Sha et al.^47^ who isolated large amounts of catalase bound to the periplasmic bacteria *Brucella abortus*.

The F0 showed the highest sialidase-specific activity in anion exchange chromatography. This is presumably due to the influence of the initial isolation method using chloroform. The use of chloroform is thought to disrupt the surface charges of NanB sialidase, resulting in the inability to bind to the Q-sepharose bead. However, this unexpected finding did not meaningfully hinder this study, as evidenced by the presence of sialidase activity shown in the F0 fraction. The molecular weight of NanB sialidase was supported by the prediction results of 3D preparation in the Raptor X program which predicts that NanB sialidase *Pasteurella multocida* 86–1913 has a molecular weight of 56.44 kDa. Previous study reported different results, which predicted the molecular weight of NanB sialidase *Pasteurella multocida* of 36 kDa^48^, 250 kDa^49^, and 500 kDa^50^.

NanB sialidase *Pasteurella multocida* showed 35℃ as optimum temperature. Slightly differing results were reported in previous studies, that the optimum temperature for sialidase from *Listeria monocytogenes* is between 30 and 35 ℃^51^. In contrast, the optimum temperatures of NanH and NanJ of Clostridium perfringens type D were found to be 43 °C and 37 °C, respectively^18^. Based on these reports, it can be assumed that different types of sialidases from different origins also have different optimum temperature.

Previous research regarding the optimum pH of sialidase from *Pasteurella multocida* had an optimum pH between pH 6.2 and 6.8^19^. However, at pH 5 and 6 in this study, sialidase activity was still found to be remarkably good. This lower pH value has previously been used as a standard by Worrall et al.^11^ in making sialidase-based vaccines. However, sialidase did not have good activity at pH below 5 and above 8. This was caused by damage to the enzyme structure due to the extreme pH (Fig. 7B).

As expected, sialidase activity decreased when incubated at 37 °C for 72 h (Fig. 7C). The decreased activity may cause by accumulated damage to the protein structure due to the natural action of proteases. However, this factor can be considered in developing sialidase as a drug, namely that the treatment process will be effective because the enzyme activity still lasts for several days, even at body temperature.

In developing new drugs in the health sector, safety is a central factor that must always be considered. The toxicity test in this study aims to determine a safe dose that does not damage red blood cells, which is an indicator for further testing. Based on the toxicity testing results, NanB sialidase has very low toxicity. The highest toxicity value was shown at 0.258 U/mL dose of sialidase, which was indicated by 2.12% lysis of chicken red blood cells (Fig. 8A) and 7.65% lysis of rabbit red blood cells (Fig. 8B). However, this dose should be tested further, considering that the target of sialidase development is epithelial cells that quickly regenerate cells if there is minor damage due to high doses of sialidase. Previous research by Larson et al.^52^ in assessing the toxicity of sialidase concluded that sialidase did not show any toxic effect on the various cell types tested. A similar conclusion also stated that sialidase was proven to be non-toxic to human respiratory organs as a therapeutic target^8^.

Rabbit red blood cells are more susceptible to hydrolysis than chicken red blood cells, even with the same α2-6 and α2-3 bonds. This is presumably because there are differences in the red blood cells of the two. Chicken red blood cells possess a nucleus, which makes chicken red blood cells more stable than mammalian cells^53^. The study by Baier et al. concluded that the type of red blood cells of each species has a difference in thickness and adhesion that affects the strength of all cell properties. In mammalian red blood cells, adhesion increased with elevated temperatures and scaled with reported membrane sialic acid concentrations. In chicken only adhesion decreased with higher temperature. Previous study showed that NanB sialidase developed recombinantly in *E. coli* cells was able to hydrolyze both types of sialic acid well, with a higher tendency and ability to hydrolyze Neu5Acα(2–6)Gal^19^.

## Conclusion

Based on the results of in silico tests and experimental studies, NanB sialidase of *Pasteurella multocida* B018 has been shown as an eligible candidate for further development of sialidase-based antivirals. Further studies are necessary to investigate whether red blood cells that have been treated with NanB sialidase of *Pasteurella multocida* B018 can still agglutinate specific viruses. Further research on the activity of NanB sialidase of *Pasteurella multocida* B018 on MDCK cells is required to explain the ability of NanB sialidase to inhibit the avian influenza virus.
